# A case report of Brugada-like ST-segment elevation probably due to coronary vasospasm: Erratum

**DOI:** 10.1097/MD.0000000000010504

**Published:** 2018-04-13

In the article, “A case report of Brugada-like ST-segment elevation probably due to coronary vasospasm”^[1]^, which appeared in Volume 97, Issue 9 of *Medicine*, affiliation a and the corresponding author's information appeared incorrectly and should be:

^a^Department of Cardiology, Beijing Friendship Hospital, Capital Medical University, Beijing, China.

^∗^Correspondence: Yongquan Wu, Department of Cardiology, Beijing Friendship Hospital, Capital Medical University; Beijing Key Laboratory of Metabolic Disorder Related Cardiovascular Disease, Beijing, China (e-mail: wuyongquan67@126.com).
